# “Get on with it. Cope.” The compassion-experience during COVID-19 in UK universities

**DOI:** 10.3389/fpsyg.2023.1112076

**Published:** 2023-06-21

**Authors:** Fiona Denney

**Affiliations:** Brunel Business School, Brunel University London, London, United Kingdom

**Keywords:** compassion, higher education, public sector, leadership, education, COVID

## Abstract

**Introduction:**

The COVID-19 pandemic caused major disruption to all sectors including higher education during the years of 2020 and 2021, thus providing a window into how different types of suffering can combine and the role of compassion in alleviating pain. Higher education within the United Kingdom provides a case example in this study, but the lessons about compassion are transferable to other contexts, particularly those in the neoliberal public sector. The impact of the pandemic period on teaching in universities has been well documented but there has been far less written about the wider experiences of staff who worked through this period, their suffering and the extent of compassion within their work lives.

**Methods:**

29 interviews were conducted and individuals were invited to talk through the story of their pandemic experiences from March 2020 to the interview date of December 2021. Storytelling is a common method in organization studies and, although research into compassion in organizations is nascent, this method has been used in other studies.

**Results and discussion:**

Previous research has examined organizational compassion in short periods of crisis and this study therefore provides a contrasting perspective on how compassion shifts over a longer period of suffering. A distinction is drawn in this study for the first time between “formalized” compassion processes in the organization which structurally prioritized compassion for students over that of staff, and “informal” compassion shown between staff to each other and between students and staff. The more that formalized compassion was evident, the less apparent it was in interpersonal interactions due to staff wellbeing being compromised and a systemic failure to recognize the dependence of student compassion on the wellbeing of staff. The findings therefore lead to theorizing that although neoliberal universities are perceived as being full of organizational neglect, compassion was structurally embedded for students but at the expense of staff.

## 1. Introduction

The COVID-19 pandemic caused major disruption to all sectors including higher education during the years of 2020 and 2021. As the world returns to a pre-pandemic way of living, it is important to harness any lessons learned during this time. The pandemic period provided a unique opportunity to study compassion in organizations as it brought to the fore enormous suffering through the illness itself combined with insights into employees' living conditions at the times of lockdowns. Kanov (2021) refers to two distinct types of suffering—that which is inevitable and experienced as part of the human experience, and that which is avoidable and can be caused by the environment within which we work. The COVID-19 pandemic provided a window into how these types of suffering can combine and the role of compassion in mediating the suffering—or in exacerbating it.

Globally, higher education at the time of the pandemic was thrown into disarray and much has been written about the immediate pivot to online teaching. This is, however, only one aspect of working in universities and less research has focused on the wider experiences of those working in higher education during 2020 and 2021. Much can be learned about compassion during this time, to identify what would be useful for the higher education sector to learn in terms of compassionate interactions between staff and the extent to which compassionate leadership is needed. As is written elsewhere (c.f. Waddington, 2016, 2021; Denney, 2020a, 2021a,b) neoliberalism and market approaches to higher education have contributed to a compassion deficit in universities and an increase in staff and student suffering. COVID-19 was a period where everyone was experiencing stress and uncertainty and where the working from home situation was less than ideal. As such, COVID-19 provided a specific timeframe within which to study compassion and to identify any changes throughout this period. The study reported here identifies how compassionate behavior changed throughout 2020 and 2021, the experiences of those working in UK higher education at the time, how they were affected by compassion, or a lack thereof, and the reasons for what was happening. This is in contrast to previous studies of compassion in crisis, where the periods of crisis have been far shorter (Dutton et al., 2006; Simpson et al., 2015). It also identifies how university leadership was perceived during the same period and the degree to which compassionate leadership was experienced by staff at the various universities in the sample.

Although the focus of this study is not on stress and work productivity, it is important to relate the experiences of those working in higher education to this wider context. Work-related stress is one of the biggest causes of employee absenteeism in the UK and the Health and Safety Executive (HSE) estimates that 17 million days of work were lost in 2021/22 due as a result (https://press.hse.gov.uk/2022/11/23/hse-publishes-annual-work-related-ill-health-and-injury-statistics-for-2021-22/). The development of neoliberal ideologies in the higher education sector has led to the application of practices more generally found in capitalist industries, with a focus on bureaucratic procedures rather than education (Bush, 2018) and a preponderance of targets, key performance indicators and market economics. Worline and Dutton (2017) identify some of the causes of workplace suffering as being underappreciated and undervalued, not having control over their workload and feeling that their work lacks meaning and these are known factors related to stress in the workplace that can impact negatively on productivity. Although academics report a reasonable level of control over their work (Kinman and Jones, 2008), the THE University Workplace Survey of 2016 found that academics feel exploited and ignored by management, thus indicating a considerable level of suffering. In any workplace context it is therefore important to understand the sources of stress and suffering and to be able to identify how compassion can help to mitigate this.

Overall, there is much that can be learned about compassionate behavior in organizations from the narratives in this study. Compassionate leadership needs to be encouraged and developed as part of management training, particularly around how communications can be perceived by staff and how they may differ from local behaviors. Individual acts of compassion between staff members and between staff and students were more common throughout the pandemic period, and universities need to take the opportunity for storytelling from this period to provide examples of how compassion can have a big impact on wellbeing and motivation. Furthermore, for the first time this study provides an insight into how compassion ebbs and flows during a long period of intense stress, particularly when there are attempts to embed compassion in policies for one community group over another. The study provides an insight into the compassionate interactions between different groups and There are currently no examples of higher education institutions providing post-pandemic opportunities for staff and students to share their experiences and recover collectively from the impact of lockdowns, yet storytelling is known to enable staff to bond together through shared emotions (Czarniawska and Joerges, 1997; Simpson et al., 2020). I therefore recommend that this would be an opportune moment for universities to invest in the mental health and wellbeing of their communities by providing opportunities to share collective experiences in psychologically safe spaces and thereby contribute to the creation of healthier universities for the future.

## 2. Background

### 2.1. UK universities prior to COVID-19

Prior to COVID-19, UK universities, in common with many of their counterparts in other developed countries, were experiencing enormous challenges as a result of being part of a globally marketized environment. In the UK, there were moves toward making higher education a free(ish) market from the 1960s onwards (Middlehurst, 2004) although many changes were as a result of the Dearing report in 1997 (Dearing, 1997) leading to considerable expansion of the numbers of UK higher education institutions and ultimately resulting in the management of universities as global businesses (Deem, 1998). Subsequent changes to the funding systems such as the introduction of a blanket £9K fee for home students and reductions in direct government financing has led to shortfalls between costs and income. This combined with the business model has led to many universities increasing student numbers but not staff at a comparative rate. More staff are employed on fixed term, temporary and even zero-hours contracts leading to mass casualization across the sector. These staff were the first casualties of COVID-19 as many universities abruptly terminated their employment as soon as the effects of the pandemic were felt in the UK (Denney, 2020a,b). At the same time as changes in funding, the UK government established a sector regulator, the Office for Students, which has in turn substantially increased the role of scrutiny and external assessment. Together these factors have created a pressure-pot for staff within the system and academics are increasingly experiencing work-related stress (Erickson et al., 2020) whilst there is, at the same time, no evidence that the students' educational experiences have been improved. A key issue at the time of COVID-19, therefore, was *at what cost are we prepared to continue to cause suffering in our higher education universities and system?* If the answer is to continue with the suffering within the system, the next question is then *what can compassion teach us in order to help redress the balance and reduce suffering?*

### 2.2. Compassion

Compassion in the field of organization studies is relatively recent although burgeoning, since Frost (1999) highlighted the extent of suffering at work and the importance of developing compassionate responses. Compassion, although often misunderstood colloquially as an emotion, has been defined as an action to ameliorate suffering (Worline and Dutton, 2017), and a four stage process has been identified by both Worline and Dutton (2017) and Simpson et al. (2019), consisting of the following:

1. *Noticing* that suffering is present;
2. Making *meaning* of the suffering in order to create the desire to alleviate it;
3. *Feeling* empathic concern for those who are experiencing the suffering;
4. *Taking action* to alleviate the suffering to some extent at least.

Compassion in the workplace is associated with better well-being and health of employees and a positive relationship between colleagues, as well as better bottom-line results. Compassion has also been linked with other factors such as improved employee engagement and hence performance and positive recruitment and retention (Lilius et al., 2003; Poorkavoos, 2016; Guinot et al., 2020; Ramachandran et al., 2023).

### 2.3. Suffering

The compassion literature identifies two types of suffering—inevitable and avoidable. Inevitable suffering is that which comes as part of the human existence through things like bereavement and illness. Inevitable suffering happens to most people at some point in their lives and there is a role for understanding that suffering transcends any such work-life balance as might purport to exist (Frost, 1999; Kanov, 2021). COVID itself taught us this when people were working from home and juggling multiple demands just to keep a sense of normality going, to deliver on work-requirements and look after elderly family members and provide a semblance of home-schooling. Most people during COVID were suffering, even if they were not ill with the virus themselves and this has gone largely unacknowledged in the higher education sectors.

Avoidable suffering, however, is that caused within our organizations, or at least, exacerbated by them. Frost (2007) and Kanov (2021) refer to this kind of suffering as being *preventable*, in that if our organizations themselves were better designed and work structures and leaders paid more attention to how people are affected within the workplace, then much suffering caused by systems, processes and working conditions could be eliminated, or substantially reduced (Frost, 2007; Kanov, 2021). Workplaces, however, rarely invest time in listening to the stories of those within the organization to hear the honest experiences of life and work, as recommended by Worline and Dutton (2017), with a view to understanding how things can be improved (Worline and Dutton, 2017). One of the purposes of the study reported here, therefore, was to give participants the opportunity to reflect on their working experiences during the COVID-19 pandemic and to illuminate the aspects that caused suffering and where compassion was able to alleviate the suffering.

### 2.4. Compassion and crisis

Whilst suffering is part of the landscape of working in organizations, additional suffering can be caused by crisis events such as natural disasters and accidents, and organizational responses to these provide fertile ground for compassion researchers. Dutton et al. (2006) and Simpson et al. (2015) both conducted research into unforeseen crises and the responses that were experienced within organizations–one, in the Dutton et al. (2006) study, a top-tier university. The wide-ranging impacts of the recent COVID-19 global pandemic have refocused attention on the role of compassion within organizations, and on compassionate leadership, conceptualized as a style by Ramachandran et al. (2023). Leadership itself has been widely studied and is broadly conceptualized as a process, a trait, or a behavior. Contingency theory, however, probably holds the most intuitively appealing explanation that leadership effectiveness depends on the leader, their followers and the context or situation that they are operating in at any given point in time (Manning and Curtis, 2012). Leadership effectiveness, of course, is itself open to interpretation dependent on the audience asked for their point of view. If the contingency theory is taken, however, then crises are of particular interest in revealing leadership behaviors and the extent of compassion employed. COVID-19 furthermore is an even more interesting example as the crisis was far from a one-off event and was extended over a long period of time. Although Simpson et al.'s (2015) work looked at the impact of a crisis over a longer period than Dutton et al. (2006), the extent of the crisis did not last as long as COVID-19. COVID-19 therefore gives us a particularly interesting insight into what happens to compassion within organizations over a long-drawn-out crisis.

### 2.5. COVID-19 and the higher education sector

In December 2019, the World Health Organization was notified of a novel coronavirus in China, although it is thought that what became known as SARS-CoV-2 had been circulating since November 2019. By the middle of January 2020, other countries across the world were identifying and reporting cases. By the end of January 2020, some 10000 cases were reported across 21 countries, indicating the impact of global mobility and the connectedness between different cultures (Holshue et al., 2020).

The ensuing impact on higher education was enormous. In March 2020, universities across the world rushed to put their teaching and assessments online, staff were sent home to work and many students found themselves trapped in halls of residence with limited access to food. Given the extent of campus-based activities, and therefore revenue, universities were significantly impacted financially by the ensuing lockdowns. In the UK, there were calls for students to receive tuition fee-rebates although none materialized and the UK government supported universities in continuing to charge full fees throughout the pandemic period, albeit with the caveat that quality had to be maintained (Department for Education University students and COVID-19 FAQ, 2020). There was also recognition at that time that universities were using the impact of the pandemic to invest in innovative online teaching practices—something that had long been under development in most institutions but had not previously received much impetus. Universities, as large and global organizations, can take a long time to introduce innovations and there is a tendency to revert to the “tried and tested” methods of in-person teaching with ensuing resistance to engaging proactively with online formats. Put in a situation where there was no other choice, universities stepped up where they could but there was also a prevailing view that there would be a return to “normal,” interpreted as “pre-pandemic,” and any online provision was therefore temporary.

The impact of the pandemic period on teaching has been well documented (c.f. Karalis and Raikou, 2020; Leask, 2020; Pokhrel and Chhetri, 2021; Ammigan et al., 2022; Devlin and Samarawickrema, 2022; Tomej et al., 2022) but there has been less about the experiences of staff who worked through this period. Universities are large and highly complex institutions and they do more than teach students; staff are employed in a myriad of different roles and the interpretation of the impact of the pandemic on staff therefore deserves to be more nuanced. The overall aim of the project reported herewith, therefore, is to record as many experiences as possible of what working in UK higher education has been like throughout this period—to hear the different interpretations that individuals put on their roles within the academy and to understand the impact of COVID on the weaving together of personal and professional lives. During lockdowns and school-closures, people found that their professional and personal lives could no longer be as clearly separated as they had been pre-COVID. Corbera et al. (2020) comment that the confinement was not of our own making but identify that we have all had a choice in how to respond to it and it is therefore important to understand how different people make sense of the situation and dealt with the stresses that it induced. This was also a time of profound complexity for university leadership. During the period there was a very prominent focus on “students first” but it is also necessary to understand the extent to which this was at the expense of staff health and wellbeing. Paradoxically, the pandemic also presented an opportunity for people to share that they were suffering and opened up opportunities for compassionate responses. Compassion has therefore been used as a lens to interpret the responses in this study and to identify how compassion has manifested during the pandemic period from the staff perspective. In so doing, this paper makes two distinct contributions. Firstly, it identifies the experiences of staff working in UK universities during the COVID-19 pandemic period and interprets these experiences using a compassion lens. As such, it identifies how compassion ebbed and flowed during the pandemic period. Secondly, the role of university leadership is examined with the compassion lens and this study therefore provides crucial information for university leaders for the future on how they can deal with both avoidable and inevitable suffering.

## 3. Methods

### 3.1. Overall approach and sampling

The study reported here intended to identify and explain staff experiences of working in UK universities between March 2020 and December 2021. Participants for the research were invited via social media (Twitter, LinkedIn and a higher education Facebook group that the researcher is a member of) and a higher education mailing list. Following this, snowball sampling was then used and contacts of the researcher were also asked to pass on the invitation email to colleagues and during the interviews, participants were asked to cascade details of the research and invitations to take part to colleagues. 29 interviews were carried out across 11 different universities where individuals were asked to talk through the story of their pandemic experiences along the timeline of March 2020 to the date of the interview. Similar approaches in organizational compassion studies have been used by Dutton et al. (2006) in their examination of compassion organizing in the face of a fire and by Simpson et al. (2015) in their analysis of the compassion response by businesses in the Brisbane floods of 2011. Both studies scrutinized the individual interpretations of compassionate responses using interviews, and Dutton et al. (2006) also obtained further documentation. Storytelling is also a well established method of research in organization studies (Van Hulst and Ybema, 2020). This study therefore takes a similar approach to Dutton et al. (2006) with interviews and additional documents. The aim was to interview 30 participants in total and 35 contacts in total were made. In the end, 29 interviews were conducted before I had to cease interviewing due to a family crisis which interrupted work at that stage. Each interview lasted for an average of one hour, was audio recorded and subsequently transcribed.

The transcripts were analyzed using thematic analysis where key themes and sub-themes were identified in NVivo. The themes were then scrutinized for evidence of suffering and compassion, identifying descriptions of suffering, the varying stages of compassion and the context within which they occurred and the players in the interaction.

As participants were therefore self-selecting, interviewees were asked why they had chosen to take part to identify any bias. Most responded that they were aware from their own research how difficult it can be to get people to be interviewed and so they were taking part out of a sense of duty and solidarity to a fellow social science researcher. Only two people commented that they thought that they had a particular story to tell with regards to the pandemic, due to disability and specific circumstances.

As the purpose of the research was to capture the diversity of participant's experiences across the breadth of working within UK higher education, only broad criteria for participation were established, these being that individuals had to be from the following groups:

- Professional/administrative services
- Academic (faculty)—either teaching-only or teaching-and-research
- Research-only
- OR be a doctoral research student
- AND not restricted by whether they were full-time or part-time.

Out of the 29 participants, the majority were split between professional services roles and faculty/academic staff. Only 3 people were research-only and only 1 person was a doctoral student. It was also observed that relatively few participants were in senior leadership roles with only 2 people holding senior academic department or faculty leadership roles. Table 1 gives details of the breakdown of participants by gender and broad job role.

**Table 1:** Breakdown of participants by gender and broad job type.

**Gender-male/female (*n* = 29)**	**13/16**
Academic	14
Professional services	6
Academic-third space/professional services	3
Research (not clinical)	3
Clinical research	2
Doctoral student	1
All job types	*n* = 29

Broad job types distinguish between academic, clinical researchers, researchers in non-clinical disciplines, professional services staff and those in “academic/third space” roles. Third Space are increasingly being recognized in the literature and include staff who usually have academic backgrounds (often a doctorate) and do jobs which require this expertise but may not necessarily hold academic contracts. There is uncertainty in the sector over whether these roles should be assigned academic or professional services contracts, often related to whether the employing university is research intensive or post-1992. The literature on the increase in Third Space roles is increasing and seeks to reflect the complexity of the modern university (Whitchurch, 2006, 2008, 2012; Denney, 2020b, 2021b; McIntosh and Nutt, 2022). Furthermore, there was evidence from the interviews for this study that the importance of these roles increased substantially during the pandemic period—particularly for those in academic/educational development and digital education when the pivot to online teaching took place.

### 3.2. Procedure

Two pilot interviews helped to establish the protocol whereby participants were asked to tell their “story” of the pandemic period from March 2020, when most UK universities went into lockdown and there was an emergency “pivot” to online teaching and assessment, through to the date of the interview. The interviews were all carried out in November and December of 2021, where the UK was still in and out of lockdowns and pandemic restrictions and universities were emphasizing different balances of in-person and online teaching. Participants were asked to comment on how they interpreted university-level communications and to talk about anything that they saw as being of particular significance throughout the pandemic related to work. As such, this was a broad mandate and gave participants significant flexibility in how they identified and interpreted what they wanted to talk about. They were also asked if they had any additional materials that they would like to provide to illustrate aspects of working in the pandemic period and many did—varying from personal photographs through to podcasts and teaching materials.

The interviews were therefore loosely structured as the aim was to draw out everyone's COVID story. Interviewees were guaranteed anonymity, including assurance that their institution would not be identifiable from any quotes used. There were initial concerns that participants may be unwilling to talk about problems in their institution during the COVID period, so this was identified in the ethical approval stage, and additional statements of reassurance regarding anonymity were included in the Consent Form and Participant Information Sheet, as well as at the beginning of the interviews. A question list of probes was established to draw out factors relating to experiences of balancing work and home life, mental and physical health issues and views on university communications and leadership and management during the period. These questions are included in Appendix 1.

The interviews spanned 3 academic years–2019–2020, where the first lockdowns were introduced in March 2020; 2020–2021 where a significant proportion of the participants were still teaching online and the start of 2021–2022 from September to December. Nearly all the interviewees struggled to distinguish between the start and end of each academic year and most lost their place in the timeline at some point, which reinforces the impact of the pandemic in blurring the boundaries between the different academic years.

### 3.3. Analysis

The process of analyzing the transcripts followed an inductive approach, looking for broader themes and underlying patterns in the experiences of higher education staff during the COVID-19 pandemic. The process involved identifying themes in the raw data using a first-cycle coding approach, followed by subsequent re-organization of those codes into second order categories. The coding framework used an initial round of descriptive terms and subsequently interpretative codes and finally pattern codes (Miles et al., 2020). The coding process was iterative and further analysis of the data followed which led to the identification of the compassion phases discussed here.

### 3.4. Methodological limitations

Management research involves addressing complex phenomena and qualitative approaches to investigating these phenomena emphasize the uniqueness of the information obtained to build theory. In this study, the contribution lies in both the individuality of the experiences of those interviewed as well as the similarities which have led to the development of the themes discussed below. One of the strengths of the study is that the five phases identified in the results were discernible across multiple participants. However, these are the reports of only 29 people employed in universities throughout the pandemic period and are not necessarily representative of the wider population of staff. Additionally, accuracy of reflections vary over time and one shortcoming of this study is that it relies on the memories of the participants. Participants were asked to provide additional documentation to support their stories but not all did. The types of documentations varied from photographs to evidence of work carried out but interestingly no journal entries were provided which would have strengthened the study by allowing for an additional layer of data scrutiny.

### 3.5. Reflection

Although only 29 people were interviewed for this study, my own experiences as an academic working in a UK university throughout the COVID-19 pandemic period have no doubt played a part on my interactions with the data and in the interviews. In qualitative research, it is impossible to divorce the researcher from the research and although a systematic approach has been applied to analyzing the data, it is impossible to remove oneself completely the process. Easterby-Smith et al. (2021) refer to different perspectives on reflexivity and how the researcher seeks to be aware of their presence within the research process. Being an academic myself during COVID-19 played a strong part in my desire to conduct this research and to provide an opportunity for others to tell their stories. The interviews resulted in some intense and personal conversations, and occasionally participants would get upset reflecting back on their experiences. I was not outside of this and I shared the pain that they were talking about. When designing the research, I had perhaps not taken this into account as well as I might, and I soon realized that I needed to space the interviews out in order to give myself a break from the intensity of being back in the stress of the pandemic period. The suffering was shared throughout the interview process and it is therefore not possible to be a dispassionate observer for this piece of research. Instead, throughout the interview period, as indeed I did throughout the pandemic period, I captured my own feelings and observations in a journal. The results and discussion below therefore include my own views and also relate the findings to the wider environment within which the experiences were taking place.

## 4. Results and discussion

Analysis of the transcripts led to the identification of five discernible phases of suffering and compassion throughout the pandemic period. Whilst the timeframes of these phases are not exactly the same for all participants, they were broadly similar enough to enable them to be grouped. The phases are identified as follows:

Phase 1— March to September 2020 *Novelty Phase*
Phase 2— September to December 2020 *Compassion Focus on Students*
Phase 3— December 2020 to March 2021 *On our Knees*
Phase 4— April to September 2021 *No End in Sight*
Phase 5— September to December 2021 *Compassion Fatigue*

The following sections talk through each of the phases in turn and identifies the suffering that characterizes the phase, along with the level of compassion.

### 4.1. Phase 1—March to September 2020 *novelty phase*

Participants in this research project identified the first phase, between March and September 2020, as being a novelty phase:

I think working from home, in March… it was… there's that perception of it being fun at *first, it goes through a real rollercoaster and you're thinking, “This is only going to last two weeks, we'll be back in the office maybe in a few weeks' time.” AW1*

This was characterized as being both positive and negative in that there was a huge amount of shock and fear present:

*one of our trainees was infected with COVID-19 and passed it on to two thirds of the group, including me. BL1*

*We just kind of plodded on in a very stressed out way with the students all a bit scared, us all a bit scared, apologizing to them then saying, “You're doing your best,” and luckily I think the semester lasted about 4 more weeks and then it was the Easter break. EH1*

But it also presented a good opportunity for those who had an interest in online and digital education to try out a few things that they had wanted to experiment with:

*We wanted to maintain a practical element as well, what practicals we can do at home…I was doing…science practicals with my son… I was using his Playmobil cars, and we were making ramps. I was drawing on the ramps, distance. My son just thought it was fun, attaching some masses to the end and watching it drop and see it zoom. But I was using those photos to make a video to say, “This is how they can measure how mass affects speed.”…We did digestion. So, making poo through tights and stuff. BL1*

Or indeed felt that the initial lockdown presented the challenge of how to engage with students and responded to that creatively:

*I think it was about going out there and finding those tools and trying to make the learning different. Not trying to make it the same because it wasn't. Trying to make it different, trying to make it engaging. The other silly thing that we did, we'd start the morning session 15 minutes early, or whatever session, and we'd have music going. The students would tell us what music they wanted. So, I've got my Alexa here…I'd say to the students, “Oh, just ask it to play something.” That would start a dialogue about when they remembered that song, what they liked about that song…It was really from the beginning but then, it was picking up these tools. “How do we keep our relationship with our students going?” Because it's a really hard one and I'm a firm believer. If you don't develop that relationship with the student, then learning doesn't happen. SS1*

In the UK, universities went into lockdown and remote teaching toward the end of March when the Easter break was looming, and at a time when the majority of undergraduate teaching was coming to an end for the academic year. There were concerns about how to pivot to online assessment but the pressures were not felt to be immense at this stage, although many of the participants characterized this phase as being a “rollercoaster” of emotions. The suffering was more focused collectively and externally—this was something that everyone was experiencing together and compassion levels were very high at the time:

*We were very forgiving and our students were very forgiving because it was all new and it was all unknown. EH1*

There was also no understanding of how long the pandemic and lockdowns would last and although there was daily doom and gloom in the media, there was also quite a lot of lighthearted focus on how everyone was adapting—zoom yoga, Joe Wicks' online PE classes and pets interrupting working from home meetings. The fear was interspersed with a sense that we were all in this together and also that we had emotional resources that we could tap into in order to find compassion for others, as well as being on the receiving end ourselves. The weather in the UK was also beautiful in the summer of 2020 and many interviewees referred to the fact that everyone had to take holidays in the UK as not being problematic that year. As a result, there was a sense of vitality for the first period which doubtless helped the compassion. At this particular point, it is possible to view working from home as being a positive enabler for compassion. Many of the participants reported enjoying spending additional time with family and engaging in pursuits such as baking and board games that they had not previously had time for.

*The first bit I had my whole family home which was lovely; having 3 grown-up children move back in with you is fantastic and the weather was lovely. CG1*

*In some ways, there's been some good things. When lockdown first happened, my two daughters were away at university, and they came home. They were both here, literally in the room there, cooking constantly. Baking cakes and all that sort of stuff, which is part of the problem. There's more of me now, than there was before lockdown. Much too much more. In some ways, it was nice. My wife was home. She's a teacher. She was teaching from here. So, I would be in this room, my daughters would be in the dining room doing their university work and my wife was upstairs… When it all first kicked off, I was being quite good and going lots of walks... We've got woodland nearby and there was no air traffic or road traffic. So, all you could hear was the birdsong and that was actually really good for my head. PC1*

*And ironically I felt good because I was at home here with my family. I mean, again, not everyone, this wouldn't be everyone's experience, but I've got three kids, the oldest is 23, I've got a 21 and just about to be 18. So, the middle on was due to go to university and didn't. The youngest one was still at school so was home. And the oldest one got furloughed and came home. For the first time for years, we were together as a family, and we had lunch together every day, and I live in a beautiful part of the world. I'm looking out over trees and if I open the window I can hear the see actually, you know, and what's not to like, you know…I don't want to get starry now, but it was a glorious time actually, which actually funnily enough, my eldest one is still living at home, the other two have gone off to do things, but the oldest one was talking, and she said, “Do you remember lockdown Dad? We used to come and have lunch together every day and it was lovely, we talked about things.” NP1*

This phenomenon has also been reported elsewhere (The perfectionism trap, 2021). It is therefore hypothesized that the Resource Investment principle of Conservation of Resources theory may play a role in the experiences in this phase. Stress and loss of normal life were being experienced and people were taking the opportunity to invest in resources around them (family, exercise etc.) in order to mitigate against the sense of stress caused by COVID and loss of normal life. The slowing down of life and the ability to do this in the initial phases opened people up to being more compassionate with each other.

### 4.2. Phase 2—September to December 2020 *compassion focus on students*

The second phase coincided with the start of the new academic year in the UK. At this particular point, there was again more novelty with the focus being toward creating online teaching that was meaningful and effective for students. As a result, the focus during this time was very much on students themselves and universities were identifying ways in which they could build in compassion into their policies for students. For example, most universities produced *no detriment policies* which enabled students to make multiple claims for the impact of COVID on their assessments. Whilst this was a way in which suffering of students was recognized and alleviated, it had a negative impact on staff, who had to deal with an almost continuous marking load and felt significantly let down by management.

…*the university decided to implement some policies around submissions, where the students had the original [deadline] and then they had an extension, and then they had another extension [which]…kicked on any resubmissions. So we were finding ourselves with multiple submission points…when dissertations were due in April, some of them didn't come through until July. In fact, one didn't arrive until the end of August…I felt we were overlooked because these multiple submission points caused huge stress to staff. It meant that holiday times were interrupted. It meant for me…managing a module where there was multiple submission points, that you could never put it down…I don't feel that was well thought out, in relation to staff's wellbeing. SS1*

The compassion element at this phase is very interesting because it was institutionalized and formalized but prioritized one community group (the students) over another (the staff). In Phase 1, compassion had been managed locally and informally but the attempt to build it into institutional structures neglected to consider any unintended consequences on staff.

*I think…there was perhaps a greater sense of accountability around the student experience and around student satisfaction…To me, it felt like power and control…I think maybe part of it was we need to know what's happening because we want to make sure that we can tell other people. We need to tell governors, we need to tell society, we need to tell our region what we're doing so that everybody knows that we're doing the right thing for our students. FS1*

In terms of the 4-stage model of compassion, it seems as though UK universities had *noticed* the suffering of one group, students, and had moved through the model to *respond* by building in multiple submission points to allow for the impact of COVID on students' abilities to be assessed fairly. This is, of course, laudable and an appropriate response in the circumstances. It did, however, completely ignore the reality that staff were also suffering greatly at the time. Not only were staff suffering with higher workloads, but the impact of lockdowns meant that many were trying to juggle childcare and home-schooling with a constant demand for more and more continuous work from their employer. No wonder they felt ignored.

At the same time, some universities were attempting to get students back onto campus by introducing a more hybrid model of teaching. Again, the focus was on what would be most beneficial for students but staff found that attendance was very patchy. Given the effort put in by staff, and the fear experienced by many in returning to campus, this again was an area where *response* to student compassion outweighed considerations for staff.

*I think the students were still pretty tolerant. We did put on some in-person events…Even when we were doing all the lectures in online environments, we did have some live classes. Students said they were going to turn up and then, very few did, actually…It was a bit disappointing, but you can't really blame the students. PC1*

Although many of the decisions about how to teach were left up to individual departments which did allow them to take into consideration the needs of individual staff, some universities did try to implement a return full-scale to face-to-face teaching. This was a decision taken by senior leadership which brought them into conflict with the localized consideration of staff needs – an apparent clash between institutionalized compassion for students and localized, informal compassion for staff.

*In September 2020 the university decided it would return, face-to-face and at that point, I just went, ‘No'…But my line manager… was absolutely superb and we agreed that everything I would do would be online. There would be no face-to-face…I chose to keep completely away because they were in contact with students and I didn't want to put myself in any risk…By Christmas, the university had decided they were going to have to go back because Christmas [2020] was disastrous… SS1*

Phase 2 is therefore characterized by a mix between institutional policies aimed at formalizing compassion for students such as the *no detriment* policy and not only a lack of for staff, but an exacerbation of staff suffering due to it. In some cases, this was mitigated by local line management compassion, as in the example given by SS1 above. For other people this was more problematic where local line management was intent on implementing institutional policy. The interviews demonstrated that there was definite patchiness across the sector in terms of how institutions approached this issue – some participants reported feeling well supported by their institution to carry on working remotely, whereas others expressed quite strong emotions about how management treated staff at this time.

…*colleagues and my team and the people I work with were fantastic and so supportive but we had to build our own…support groups…institutionally we got four days extra leave throughout the year…but no change to workload allocation…it's quite eye-opening how a senior executive made decisions on like gut and whim and brain farts without any evidence or any proper consultation has been quite shocking to be honest. SZ1*

This period tells us a lot about apparent clashes of compassion in our organizations and something that needs to be learnt is that attempts to introduce compassionate policies for one group in our organizational community can have a knock-on detrimental effect on others.

### 4.3. Phase 3—December 2020 to March 2021 *on our knees*

Phase 3 was identified as being a time of contradictions. The Christmas period in the UK that year had been quite brutal in terms of lockdowns and COVID restrictions, so staff did not feel that they got much of a break. As a result, they did not return to university in the January feeling at all refreshed. At the same time, however, they were now familiar with the practices of teaching and working online and this specifically was no longer causing large amounts of anxiety and stress. These working and teaching practices were no longer a novelty however, so any energy from that sense of newness was now lost. It was also during this period, that there were a number of communications from the Minister of State for Universities, Michelle Donelan, that were causing senior management to behave more aggressively toward staff. On 30th December 2020, right in the middle of the university Christmas break when most institutions were closed and staff were on leave, she issued a letter to all students advising them that from the end of January 2021, there would be a phased return to in-person teaching on university campuses. This was accompanied by a letter to University Vice-Chancellors and in some areas, was taken as an opportunity to start to force a return to campus without due consideration of what staff were experiencing at the time.

*I think there were a lot of staff who were very worried about face-to-face…A lot of academics are over 50…Some, a little larger than we should be, especially after lockdown and then there's all the other health conditions and maybe have got children and complex lives as well. I think a lot of people were reluctant to do face-to-face, which I understand. PC1*

*Christmastime last year[2020], one colleague called me, like, she texted me on Friday night, 11:00 p.m. saying, “Can we talk at some point?” and I was like, “Okay, what's going on, is everything okay?” and so I called her back and she didn't pick up the phone and she [said]everything is okay, I will give you a call on [another day]. Okay, and then I was worried a little bit and I was concerned about this and I called her back on Saturday morning, so the day after and after a couple of minutes of hello, how are you and that, then and she broke into tears and like she would, it was…a lot like crying for like sad, a sadness or, it was like hysteric….I mean, she told me that she would be most clear that she reached a point where, you know… RN1*

University senior leadership in many institutions had tried to support staff by closing for 2 weeks over the Christmas period of 2020/21 with the view that this would give staff a much-needed break, even if they could not travel. As illustrated by the quote above from RN1, staff were not just exhausted – in some cases they were near breaking point. The timing of the mandate from Michele Donelan regarding a return to campus could not have been worse, and could actually be regarded as being punitive to staff who had also suffered enormously during the lockdowns.

This phase is characterized by a clash between government policies which were putting senior leadership in universities in a difficult position, the fear of some staff regarding a return to face-to-face teaching and the fatigue experienced by everyone.

*I think, by then, the students were more fatigued by the whole situation. I think a lot of them thought, ‘Well, you should have fixed it by now.' I can absolutely get that. It's not their fault. They are the victims in this… They're worried, they're uncertain, their futures…Will they get a degree that's worthwhile?...All this sort of stuff…But yes, I think the students were still pretty tolerant. PC1*

Some returns to campus did therefore take place in the January of 2021.

### 4.4. Phase 4—April to September 2021 *no end in sight*

Phase 4 was characterized by even more exhaustion. At this point, the academic year should have been coming to an end and staff should have been able to take much needed holidays. Unfortunately, the compassion policies implemented for students were having a further knock-on effect on staff at this point in the academic year because again so many students were able to undertake resubmissions and submit work at multiple submission points. For the second academic year running, the 2020–21 academic year ran straight over into the 2021-22 year without any break at all. Whilst staff were appreciative of the concerns for students, it was, without doubt, at the expense of staff health and wellbeing. Furthermore, whilst some international travel had opened up again and restrictions were gradually being lifted, the weather in the UK was not as nice as it had been in 2020. Any holidays that staff were able to take were therefore not as pleasant as they had been the previous year.

*I think that year, 2021, for staff, was a tough year…I think there's been a larger number of people with mental health problems. Problems with isolation, anxiety because those members of staff who were having to be the responsible person for all the students, have got all the stress that everybody else was experiencing as well. PC1*

The summer was also marred by poor results in the annual National Student Survey—a measure of student satisfaction that is used heavily in league tables and to provide national information about the quality of higher education in the UK. The COVID experience meant that students were using the survey to express their frustration about lockdowns and institutional policies for the most part, but academic staff experienced it as something quite personal.

*My initial feeling was “That is a massive kick in the teeth from the students that we've worked so hard to support”. PC1*

### 4.5. Phase 5—September to December 2021 *compassion fatigue*

Probably unsurprisingly the final phase identified in this study is characterized by exhaustion and fatigue, affecting the interactions between staff and management and resulting in the perception that management no longer had any compassion for staff at all. The first term of the 2021–22 academic year was an experiment in hybrid teaching for many, which combined both in-person and online teaching simultaneously. Universally this was loathed as staff found it impossible to interact with students present in-class in front of them at the same time as responding to chat messages and online interactions on the screen:

*I think the danger is by trying to help everybody you can make it a worst experience for everybody. That's my fear, yes. And I also don't think having been very supportive with the university and its management of this, I think there are some issues now which the managerial team who haven't experienced much work on the ground with this are not understanding. So, the idea of hybrid is so tempting financially, student numbers, you know, I don't need to say all those things to you, you can see why it's so tempting, but if you haven't actually tried it, you don't really realize how difficult it is. So, I think there is a misunderstanding around that. NP1*

There were some quite aggressive moves by management to get staff back onto campus in person, with no apparent acknowledgment of what staff had gone through in the preceding eighteen months or any concern about whether they had anxiety and fears about returning to in person interactions, or any consideration of underlying health conditions.

*Going back into the unit for me, was a mammoth step…I had to manage that in a very careful way, by going in and walking around. Going to my office, meeting a colleague for a cup of coffee. Just generally, getting used to being on campus again. I felt very vulnerable, extremely vulnerable because the government had taken off, no masks, no social distancing. So, students are walking around without any masks on…And I'm the person that still hasn't been into [supermarket]. I'm still doing online shopping…I can only liken it to when you've been off work for an illness and then you go back. Everything's faster, everything is in your face, everything's noisier and trying to find your way around systems. SS1*

Some of the messaging from management was seen as being inflammatory and tone-deaf to staff experiences.

…*you've put your finger on the bone of contention there…the university message is we are teaching face-to-face. NP1*

Teaching staff also referred to frustrations when they were being asked to undertake occupational health assessments concerning any underlying conditions which made a return to face-to-face teaching risky which were then subsequently ignored by managers. At the same time, some institutions imposed significant health and safety requirements to enable the teaching to take place but this put an additional burden on academic staff.

*So students in order to come onto campus had to prove to us [academics] they had done two lateral flow tests a week…the university [said] “we lecturers need to see them as they walk into the classroom”…I did not get a job as an academic to be a police person…*

*The other thing we were told to do…was take temperatures of students before they walked into the room. LN1*

Staff were also frustrated by the focus on in-person teaching. Several participants pointed out that they had been doing online teaching before COVID because it was pedagogically appropriate and felt that the blanket policies being implemented by their institutions were therefore inappropriate and a retrograde step. This was seen as a fear response to pressure from government, as opposed to an opportunity to improve further blended and online offerings to students.

Most institutions implemented some form of hybrid teaching in order to accommodate the varying needs of students such as those with underlying health conditions or caring for vulnerable relatives, as well as to allow for international students who were not allowed to travel to the UK by their own country at that time. Unfortunately hybrid was universally hated by everyone in the sample. In addition, participants referred to the extent of the fatigue that staff were experiencing and the clash between staff fatigue and student expectations.

*I think staff are tired. They started the year tired…I think the students have come back expecting more normality than is possible…There's a little bit of unhappiness that we're not fully back 100% face-to-face. They kind of understand it but they're unhappy about it as well. PC1*

Staff were still trying to demonstrate understanding of, and empathy for, the position of students at the time, but from a position themselves where their own emotional resources were completely depleted.

Some universities in the sample appeared to be taking a more softly, softly approach where they tried to encourage staff back onto campus but were not forcing them. Collectively though, several participants referenced that they had noticed a number of colleagues handing in their notice and choosing to resign in the first waves of what has become known as *The Great Resignation*.

*Speech and Language, half the staff left. Nursing, two thirds of the staff left…And the response to that [from senior management] is, well, people need to be able to choose to leave. And I call it the canary in the coalmine, they keep replacing the canaries. LN1*

Ultimately, the fatigue meant that some staff just did not want to keep going on, and sadly compassion was fading fast.

What struck me whilst I was researching this study was the extent to which staff put students first. Although staff were not themselves experiencing much in the way of institutionally-led compassion, they were still concerned about the welfare and educational experiences of their students and there was a high level of continuous concern as a common thread throughout all of the interviews. There was enormous disconnect between what staff were experiencing from their universities and what they were giving out to their students. The following section reflects on the findings from the interviews in the context of compassion and what organizations should learn going forwards.

## 5. Conclusions and recommendations

### 5.1. Compassion—What the pandemic has taught us

If compassion is a reaction to suffering, then suffering must exist first in order for compassion to be needed. During the pandemic period in this study, from March 2020 to December 2021, university staff and students experienced suffering in the same ways as the rest of the world, and it is important to understand how the structures, processes, and behaviors in universities at the time made it either easier or harder to express and experience compassion. The experiences of the people interviewed for this study made it clear that UK universities structurally prioritized compassion for students over that of staff. On the one hand, this is understandable given that students are, in essence, paying consumers of higher education in most of the UK (with some exceptions in Scotland). On the other, a failure to recognize the fact that the effective delivery of higher education is dependent on the wellbeing of staff is a failure of the whole system. And certainly, a failure of organizational compassion.

Worline and Dutton (2017) note that compassion is often ignored in organizations such as non-profits due to a lack of resources, large workloads, additional pressures and demands for changes to make the business more efficient. All of these are present in profit-making organizations as well, but the assumption that non-profits *should* be focused on humanistic concerns somehow makes the absence of compassion much starker. Public universities in the UK are non-profits but in the neoliberal environment in the public sector they tread a fine line in a quasi-market environment where there is both substantial government funding along with a fee-paying structure for students. At the same time, resources have been cut in real terms, and the pandemic provided a perfect storm for panic over the future sustainability of universities. There was immediate mass cancellation of temporary contracts and Universities UK, representing 137 institutions, requested billions of pounds of additional financial support in April 2020, to fill losses due to the pandemic (The Guardian, 2020). The perception has therefore always been that resources in universities are highly restricted and compassion becomes squeezed out as a result.

The reality in the 21-month period under investigation here is that compassion ebbed and flowed for several reasons, including the perception of resources and the focus on putting students first. The evidence is that informal compassion was quite high at the beginning of the pandemic. Staff had personal reserves of energy and emotion from not having been through such a stressful time in the run-up to COVID. Furthermore, the novelty provided some energy and enthusiasm for trialing new ways of teaching and working and there was a lot of forgiveness on all sides as everyone grappled with learning Teams and Zoom. Interestingly though, the more compassion became formalized in the next phase, the less apparent it became in interpersonal interactions. This might partly have been due to the fact that universities prioritized compassion for students, through the introduction of *no-detriment* policies, without considering the corresponding impact on staff. Furthermore, the continued management of staff compassion was left to the individual line manager, instead of being comprehensively directed at the institutional level, as was the student approach. At best, this produced an uneven experience for staff.

Whilst universities continue to prioritize compassion for students at policy level, there was also enormous compassion for students from frontline staff throughout the pandemic period. The media representations at the time, however, portrayed a very different image, citing lazy university staff sitting at home and using old recordings of lectures. From my own experiences, those of colleagues and those of interviewees for this study, it is quite clear that the reality was completely different but the media image affected the Government rhetoric, which in turn pushed university senior leadership to bring staff and students back onto campus with, in some instances, little consideration for individual concerns. Some of the academic staff interviewed for this study reported feeling treated very badly with regards to the return to campus. In spite of all of the efforts that had been put into the teaching for students during the lockdowns—the incredible creativity and commitment of staff to make the experience as best they possibly could under the circumstances and the compassion they demonstrated for their students—the NSS results for 2020-2021 were an absolute “kick in the teeth” and staff felt badly let down by both management and the students. Compassion ebbed and flowed throughout the pandemic period, but there was no doubt that staff were on the losing side when it ebbed.

Whilst it is clear from the work of Frost (2007) and Kanov (2021) that suffering can be both inevitable (i.e., life circumstances such as ill health or bereavement) and avoidable, such as that caused by organizational practices, there is a need for all organizations not to become so focused on one stakeholder group that they cause suffering to another as a result. This is becoming too common in UK universities, and it was starkly apparent during COVID. The question remains therefore about how we can include compassion in our higher education institutions without it being at the expense of one group over another. The following section contains recommendations for universities to consider, in the face of what we have learnt from the COVID-19 pandemic period.

### 5.2. How universities can improve their compassion practices

UK universities have become, somewhat understandably, fixated by their students since the introduction of tuition fees and a quasi-market approach. The COVID pandemic period under investigation in this study tells a clear story of how universities put students first at the expense of staff to the extent that it became embedded in the institutional cultures and stories of this period (Denney, 2022). This teaches us something important about the compassion practices of organizations more generally—that compassion extended to one group in the community can be at the cost of additional suffering to another. The question therefore is how organizations can avoid this happening and improve their compassion practices.

Four-stage models of compassion from Worline and Dutton (2017) and Simpson et al. (2019) take us through a structured approach of: initially becoming aware of suffering; feeling empathy with those suffering; assessing the suffering and identifying what action can be taken and subsequently taking action to alleviate the suffering. This is not a process that can be rushed, and one of the lessons that we need to take away from the COVID-19 pandemic is that if the response involves organizational processes or structural changes, then this needs to be further evaluated in the light of the experiences of other community groups. It is therefore recommended that organizations carry out a *compassion impact assessment* in the same way that they would an equality impact assessment when introducing new policies. The questions they should be asking are:

- What is the problem that we are seeking to solve?
- Does the new policy constitute an appropriate response to the suffering of that particular group?
- Are there any other factors that we need to consider when evaluating the response to the suffering?
- Does the proposed response impact on the work and experiences of other groups in the organizational community?
- If yes, is the likely impact going to cause new or increased suffering to those groups?
- If yes, what can be done to alleviate or respond to that suffering?
- Does that additional suffering and the proposed responses mean that it is not worth pursuing the original policy?
- What is the risk/benefit analysis of the original policy following these further considerations?

Even in the height of an unprecedented situation such as the pandemic, universities still should have taken time to consider the impact of introducing policies which alleviate the suffering of one group at the expense of suffering of another. And if nothing else is learnt, this is very much a lesson that should be taken forwards by all organizations.

There is one further action that can be taken by organizations to improve their compassion practices and that is the opportunity for storytelling. Storytelling talks to an evolutionary part of our human experience in that it is the way in which groups of people have passed down information for thousands of years. Oral storytelling was a core part of tribal life before the written word became dominant, yet it is not something that plays much of a role at all in most organizations in spite of the fact that it remains a powerful form of transmission of information and emotions. Furthermore, shared stories build cultures. common identities and histories for organizations thus enabling people to acknowledge suffering and develop appropriate compassionate responses as a community (Czarniawska and Joerges, 1997; Simpson et al., 2020). It is surely, therefore, time for organizations to create safe spaces for their communities to share their stories, and an appropriate place to start would be the experiences of the pandemic period. This was a starting point for me for this study and I have done my best to provide opportunities for those working in UK universities throughout the pandemic to tell their stories. Inevitably this introduces an element of self-selection bias to the data, but it was interesting to observe how keen people were to tell their pandemic stories, thus adding further credence to the point that this is not something that universities, or organizations more generally, are offering their employees opportunities to do.

Suffering and compassion are shared experiences—they do not exist in isolation. If organizations are able to provide safe and contained spaces for the sharing of stories, suffering will be shared instead of hidden and compassion will be provided with a better opportunity for flourishing. And the more compassion we have in our organizations, the less toxic they will be for everyone.

## Data availability statement

The raw data supporting the conclusions of this article will be made available by authors, on specific request, without undue reservation.

## Ethics statement

The studies involving human participants were reviewed and approved by College of Business, Arts and Social Sciences Research Ethics Committee, Brunel University London. The patients/participants provided their written informed consent to participate in this study.

## Author contributions

The author confirms being the sole contributor of this work and has approved it for publication.

## Appendix 1 – Interview probes

1. What were your working practices like when covid hit the higher education sector in March 2020 and how did they change at that point? Could you describe for me a typical working day before covid and then during the pandemic period?
2. How did your working practices evolve over the 18 months between spring 2020 and autumn 2021?

a. Probe specifically with reference to teaching/research/admin practices

3. What would you identify as being particularly good or bad during that period–in terms of working practices? Any particularly low or high points?

a. Probe specifically with regards to balance of domestic responsibilities and work

4. Are there any people, events or things that you would identify as having been particularly significant during the pandemic period? How?
5.Do you have any journal entries, photographs or anything else that you would like to show me and talk me through, to illustrate what this period was like for you, work-wise?
6.Looking back over this period, what would you liked to have been different?

a. Probe in terms of support from colleagues, employer etc

7. Looking back over this period, what was the biggest surprise to you?
8. And what advice would you give to individual universities or the sector as a whole about working practices going forwards from covid?
9. How do you see yourself now? Has your perception of yourself with regards to work changed due to the pandemic?
10. Is there anything else that you think might be of interest for me to know?
